# Lifestyle factors as mediators of area-level socio-economic differentials in cardiovascular disease risk factors. The Tromsø Study

**DOI:** 10.1016/j.ssmph.2022.101241

**Published:** 2022-09-24

**Authors:** Sweta Tiwari, Ester Cerin, Tom Wilsgaard, Ola Løvsletten, Inger Njølstad, Sameline Grimsgaard, Laila A. Hopstock, Henrik Schirmer, Annika Rosengren, Kathrine Kristoffersen, Maja-Lisa Løchen

**Affiliations:** a Department of Community Medicine, UiT the Arctic University of Norway, Tromsø, Norway; b Australian Catholic University, Melbourne, Australia; c Institute of Clinical Medicine, Campus Ahus, University of Oslo, Lørenskog, Norway; d Department of Cardiology, Medical Division, Akershus University Hospital, Lørenskog, Norway; e Department of Molecular and Clinical Medicine, Institute of Medicine, Sahlgrenska Academy, University of Gotenburg, Gotenburg, Sweden; f Department of Health and Care, Tromsø Municipality, Tromsø, Norway

**Keywords:** Area level socio-economic status, Cardiovascular disease, Risk factors, Mediation

## Abstract

**Introduction:**

Cardiovascular disease (CVD) is a leading cause of death and disability and living in areas with low socio-economic status (SES) is associated with increased risk of CVD. Lifestyle factors such as smoking, physical inactivity, an unhealthy diet and harmful alcohol use are main risk factors that contribute to other modifiable risk factors, such as hypertension, raised blood cholesterol, obesity, and diabetes. The potential impact of area-level socio-economic status (ASES) on metabolic CVD risk factors via lifestyle behaviors independent of individual SES has not been investigated previously.

**Aims:**

To estimate associations of ASES with CVD risk factors and the mediating role of lifestyle behaviors independent of individual-level SES.

**Methods:**

In this cross-sectional study, we included 19,415 participants (52% women) from the seventh survey of the Tromsø Study (2015–2016) (Tromsø7). The exposure variable ASES was created by aggregating individual-level SES variables (education, income, housing ownership) at the geographical subdivision level. Individual-level SES data and geographical subdivision of Tromsø municipality (36 areas) were obtained from Statistics Norway. Variables from questionnaires and clinical examinations obtained from Tromsø7 were used as mediators (smoking, snuff, alcohol, and physical activity), while the outcome variables were body mass index (BMI), total/high-density lipoprotein (HDL) cholesterol ratio, waist circumference, hypertension, diabetes. Mediation and mediated moderation analysis were performed with age as a moderator, stratified by sex.

**Results:**

ASES was significantly associated with all outcome variables. CVD risk factor level declined with an increase in ASES. These associations were mediated by differences in smoking habits, alcohol use and physical activity. The associations of ASES with total/HDL cholesterol ratio and waist circumference (women) were moderated by age, and the moderating effects were mediated by smoking and physical activity in both sexes. The largest mediated effects were seen in the associations of ASES with total/HDL cholesterol ratio, with the mediators accounting for 43% of the observed effects.

**Conclusions:**

Living in lower SES areas is associated with increased CVD risk due to unhealthy lifestyle behaviors, such as smoking, alcohol use and physical inactivity. These associations were stronger in women and among older participants.

## 1 Introduction

Non-communicable diseases (NCDs) are one of the main causes of premature mortality in high-income countries, such as Norway (Norwegian Institute of Public Health, 2021). Among the NCDs, cardiovascular disease (CVD) accounts for the highest number of deaths worldwide (Balakumar et al., 2016; Benziger et al., 2016). Lifestyle behaviors such as smoking, harmful alcohol use, physical inactivity and unhealthy diets are the main risk factors that, in turn, contribute to other modifiable risk factors for CVD, such as hypertension, raised blood cholesterol, diabetes, and obesity (Balakumar et al., 2016; Benziger et al., 2016; Yusuf et al., 2004).

A vast body of research has shown that individual-level socio-economic status (SES) indicators, such as educational attainment, employment status and household income, are strong correlates of CVD and related lifestyle behaviors (Beauchamp et al., 2010; Eggen et al., 2014; Ernstsen et al., 2012; Lindroth et al., 2014; Rosengren et al., 2019). For example, in high-income countries, the prevalence of CVD is higher among individuals with low income who are more often exposed to tobacco, harmful alcohol use, physical inactivity and an unhealthy diet (Benziger et al., 2016). Some behaviors, mainly smoking and excessive alcohol consumption, are important intermediary factors on the causal pathway between low SES and poor health (Erasmus MC, 2007). However, CVD is not only influenced by individual-level factors. Previous studies have shown that the physical and social characteristics of places where people live also influence CVDs (Diez Roux et al., 2001) as well as CVD-related lifestyle behaviors (Cerin et al., 2017; Cerin & Leslie, 2008) and cardiometabolic risk factors (e.g., obesity) (Ludwig et al., 2011). One of these characteristics is area-level socio-economic status (ASES), which has been found to impact on CVD and related risk factors independently of individual socio-economic standing (Cerin & Leslie, 2008; Diez Roux et al., 2001; Ludwig et al., 2011; Smith et al., 1998).

Neighborhood effects have been described in the literature as the characteristics and behaviors of individuals living in the same areas influencing residents' behavior and wellbeing (Durlauf, 2004; Roosa & White, 2014). Galster has given 15 potential causal pathways leading to neighborhood effects, which are grouped into four categories: social-interactive mechanisms, environmental mechanisms, geographical mechanisms and institutional mechanisms (Galster, 2012). Social interactive mechanisms seem particularly relevant to the population examined in this study, with the majority living quite concentrated in town center and suburbs close by. By social interactive mechanisms, we refer to social contagion (an individual's behaviors, aspirations and attitudes changing as the result of social contacts with neighbors), collective socialization (individuals altering their behavior to conform to local social norms) and social network (individuals being influenced by interpersonal communication of information and resources which are transmitted through neighbors) (Galster, 2012). According to another theoretical model of neighborhood effects on health, neighborhoods are connected to disease and ultimately mortality through four pathways: health policy and health resources, health behavior, perceptions of neighborhoods and the physical quality of an area (Meijer, 2013) (see supplementary material page 8 for a description of the model). In this study, we focused on the second pathway from Meijer's model-namely, how neighborhoods may affect health behaviors that are closely linked to disease and mortality. This is the best studied pathway (Meijer, 2013). We estimate the associations between ASES and individual health outcomes and examine the extent to which these associations can be explained (i.e., are likely mediated) by individual health behaviors such as smoking, alcohol, snuff, physical activity, and diet.

Norway is one of the most advanced welfare countries in the world, but an intimate link between SES and health still exists. The prevalence of illness, such as CVD, and life expectancy varies by area of residence, educational attainment, living conditions and income (Dahl et al., 2014; Norwegian Institute of Public Health, 2018). Some Norwegian cohort studies have found differences in lifestyle behavior based on educational attainment (Eggen et al., 2014; Ernstsen et al., 2012). Various studies have also found associations between ASES and lifestyle factors (Global Burden of Disease 2015 Obesity Collaborators, 2017; Kleinschmidt et al., 1995; Yen & Kaplan, 1998). In the Norwegian population-based Tromsø Study, differences in risk factors were found across residential areas within the Tromsø municipality in 1974 (Thelle et al., 1976), and in 2016 (Hopstock et al., 2019; Sari et al., 2021).

There are few studies which clearly link ASES to metabolic CVD risk factors. However, the role of mediators is not clearly understood, and only few studies have identified lifestyle behaviors as mediators of the association between ASES and CVD (Saghapour et al., 2021; Zhang et al., 2021) but not metabolic CVD risk factors. To our knowledge, the potential impact of ASES on cardiometabolic risk factors via various lifestyle behaviors independent of individual-level SES has not previously been investigated. This study aimed to contribute new knowledge on the associations of ASES with cardiometabolic risk factors independent of individual-level SES, and the mediating role of lifestyle behaviors on these associations in a Norwegian general population. In addition, we did not only examine lifestyle behaviors as single, separate mediators but also the combined mediating effects of lifestyle behaviors in the associations. Given that lifestyle behaviors are often correlated (e.g., physically active individuals tend to have a healthy diet and be non-smokers), it is important to examine their independent contributions to other CVD risk factors. Furthermore, these multiple-mediator analyses allow estimation of the total contribution of all examined lifestyle behaviors to the associations between ASES and other CVD risk factors. Also, unlike previous research, this study was conducted in a municipality with a rather large geographical area but with the majority living quite concentrated in town center and suburbs close by. Finding between-area differences in health outcomes and health-related behaviors within such a geographical context would have important implications for the formulation of policies and interventional strategies on a larger scale. The findings from this study can be useful in developing area-oriented health promoting strategies in the municipality. In addition, understanding the causal pathways can help targeting the most important mediators in the association during planning and policy making.

## 2 Methods

### 2.1 Study population

The Tromsø Study is a population-based study in the municipality of Tromsø, Norway (Jacobsen et al., 2012) with seven surveys conducted from 1974 to 2016, referred to as Tromsø1-Tromsø7, to which total birth cohorts and random population samples were invited. In total, 45473 individuals participated in one or more surveys. Data collection included questionnaires, biological sampling, and clinical examinations.

**Fig. 1:**
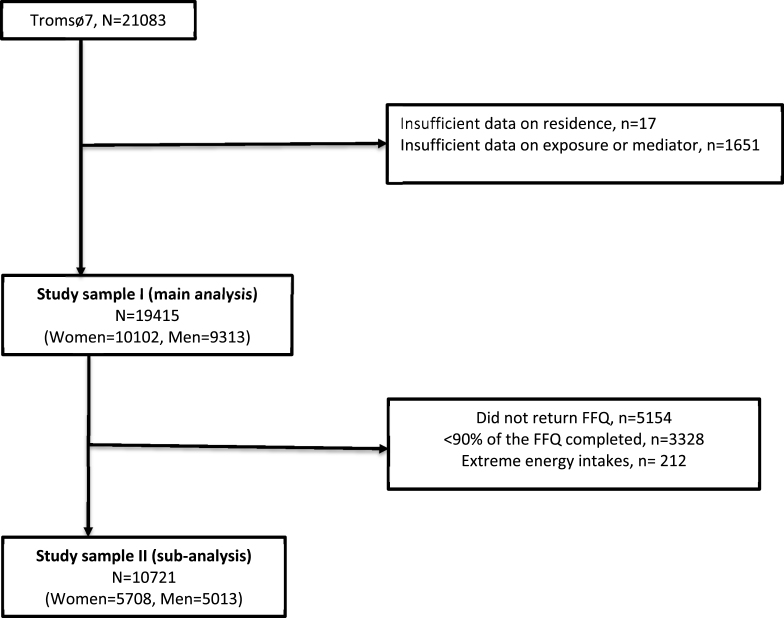
Study sample, The Tromsø Study 2015–2016. FFQ: food frequency questionnaire.

This analysis includes data from participants attending Tromsø7 (Hopstock et al., 2022) conducted in 2015–2016 (Fig. 1). In Tromsø7, all inhabitants aged 40 years and older were invited and 21083 (65%) participated. We excluded participants with missing values for exposure and mediators (n = 1668) leaving 19415 women and men for the main analyses. In a sub-analysis we included participants with valid data from the Tromsø7 food frequency questionnaire (FFQ). We excluded participants with incomplete FFQ (<90% completion) and those with unrealistic energy intake/extreme energy intake (>21267 or <3948 kJ/day) in accordance with Lundblad et al. (Lundblad et al., 2019), leaving 10721 women and men for the sub-analysis. The Tromsø Study complies with the declaration of Helsinki and has been approved by the Regional Committee for Medical and Health Research Ethics (REK), the Data Inspectorate, and the Norwegian Directorate of Health. All participants provided written informed consent. The current study was approved by REK North (reference 132624) and evaluated by the Norwegian Centre for Research Data.

### 2.2 Study area

Tromsø municipality in Troms County covers a rather large geographical area (2521 square kilometers) and is the largest urban area in Northern Norway. The municipality has a total population of 72,066 as of 2015 and the majority lives quite concentrated in Tromsø city center and suburbs close by, and only 20% lives in rural areas. Therefore, the geographical proximity between high and low SES is very small, particularly in the urban part of the municipality. A description of the areas is given in the supplementary material (page 2–4). Tromsø municipality was divided into subdivisions as per previous reports from Tromsø municipality on living conditions and public health (Hopstock et al., 2019; Tromsø kommune, 2019). Tromsø municipality defined these areas based on homogenous building type/living environment as well as number of residents. The geographical units were created so that they are sufficiently large to produce statistically meaningful results (Tromsø kommune, 2012). This division is meaningful in terms of planning and policymaking for the municipality. A previous public health report from Tromsø municipality identified large differences in health risk factors between these areas (Hopstock et al., 2019). The findings from this report were interesting, as striking differences were found among areas which are situated very close to each other. Population mobility across these areas is not high. Even in the areas with greater internal migration, immigrants have moved from outside the municipality rather than from other municipality areas (Tromsø kommune, 2019). The proportion of immigrants across areas are presented in the supplementary material (page 4). As, in Tromsø, ASES is quite stable over time, population mobility across areas is low and the effects of environmental exposures on CVD are likely cumulative and develop across many years, we performed a cross-sectional rather than longitudinal analyses to estimate the associations between ASES and CVD risk factors.

### 2.3 Area-level socioeconomic status (exposure)

The data for all individual-level SES variables and geographical areas of Tromsø municipality (36 subdivisions, hereafter referred to as “areas”) (2015) was sought from Statistics Norway, which provides official statistics for Norway. Individual-level SES variables included individual and household income, educational attainment, and house ownership. Individual income includes income from work, cash for care and parental benefit. Household income is the total household income after tax. Education was categorized into 5 levels (coded from 0 to 4) including: unknown or no education, primary/secondary school, upper secondary or vocational, university college education less than 4 years, and university college education 4 years or more. House ownership was categorized as rented or owned housing coded as 0 and 1, respectively. Standardized individual-level SES variables were summed to obtain a composite individual-level SES index. We then calculated the mean Z-score for each area (subdivision) separately to obtain an overall index for ASES. The range for ASES was −1.5 to 1.1. Higher values reflected higher ASES.

### 2.4 Baseline characteristics (outcomes and mediators)

Questionnaire data from the Tromsø Study was used for information about smoking (never/previous/current/occasional), snuff use (never/previous/current/occasional), alcohol use, leisure-time physical activity level, anti-hypertensive medication use (no/yes), and diabetes (no/yes). Alcohol use was assessed as intake per drinking session with the second question of the Alcohol Use Disorder Identification Test (World Health Organization, 2001) and categorized as no alcohol/1–2 units/3–4 units/5 or more units. Physical activity level was assessed using the Saltin and Grimby leisure-time physical activity questionnaire (Grimby et al., 2015) and categorized as sedentary, light activity and moderate-to-vigorous activity. The FFQ was developed at the University of Oslo (UiO) to measure habitual food intake in a Norwegian population with questions including frequency and amount of intake of foods, beverages, and dietary supplements. Food and nutrient intakes were calculated using the UiO food database KBS AE14 and KBS software system (KBS, version 7.3.) (Lundblad et al., 2019). We included intakes in grams/day (g/day) of fruits, berries and vegetables, saturated fat, and sugar.

Body mass index (BMI) was calculated as measured body weight/height^2^ (kg/m^2^). Blood pressure was recorded three times with 1 min intervals after 2 min seated rest, using an automatic device (Dinamap Vital Signs Monitor 1846; Citrikon). The mean of the second and the third reading was used. Hypertension was defined as systolic blood pressure (SBP) ≥140 mmHg and/or diastolic blood pressure (DBP) ≥90 mmHg and/or use of anti-hypertensive medication. Non-fasting total cholesterol and serum high-density lipoprotein (HDL) cholesterol were analyzed using enzymatic colorimetric method. Blood sample analyses were performed at the Department of Clinical Chemistry, University Hospital of North Norway.

Lifestyle factors such as smoking, alcohol use, snuff use, physical activity level and dietary variables were used as mediators and the cardiometabolic risk factors such as BMI, total/HDL cholesterol ratio, waist circumference, hypertension, and diabetes were the outcomes.

### 2.5 Statistical analysis

Baseline characteristics of study participants are presented as means and standard deviations for continuous variables and proportion for categorical variables adjusted for age and stratified by sex and ASES quartiles. Differences between ASES quartiles were assessed by regression analysis. Directed acyclic graphs (DAGs) were created for visual representations of causal assumptions between variables (refer to supplementary material on page 9). Generalized linear mixed models (GLMMs) and generalized additive mixed models (GAMMs) with random intercepts at the area level were used to examine the confounder-adjusted associations of ASES with CVDs risk factors and related behaviors.

Mediation analysis was performed to estimate mediation and mediated moderation effects. The lifestyle factors used as mediators in the analysis were smoking, alcohol use, snuff use, and physical activity level. Dietary and nutrient variables (intake of fruits and vegetables, saturated fat, and sugar) were used as mediators in the sub-analysis. The outcomes were BMI, total/HDL cholesterol ratio, waist circumference, hypertension, and diabetes. Standardized individual-level SES variables (individual and household income, educational attainment, and house ownership) were summed to obtain a composite individual-level SES index. This index was treated as a covariate in the analysis. Age was examined as a moderator of the associations. Random slopes for age were added in the model to see if it improved the fit of the model (via likelihood ratio test) as recommended by Heisig and Schaeffer (Heisig & Schaeffer, 2019)**.** We also tested for spatial autocorrelation of residuals using Moran's I test. The results did not provide sufficient evidence of spatial autocorrelation after accounting for area-level random effects in the GLMMs and GAMMs (see supplementary material page 7). All analyses were stratified by sex.

Analyses were conducted in four steps. We first estimated the associations between ASES (exposure; X in the regression equation below) with the outcomes (Y) adjusted for age (moderator; Mod) and individual SES (covariates; Co) (total effect GLMM; Step 1).

We then added an age by ASES interaction term to the total effect GLMM to estimate the moderating effects of age (step 2). Moderation effects with a *p*-value< 0.05 were considered statistically significant.

We examined mediation effects if age was not identified as a moderator in step 2, and mediated moderation effects if age was identified as a moderator. This was done by first regressing lifestyle factors (Mediator; M) onto ASES (X), covariates (Co) and the interaction term between age and ASES (X.Mod), if it was significant in step 2 (GAMM; step 3). The mediators (smoking, alcohol, snuff, and physical activity) had three or more ordered categorical levels. To establish whether they could be modelled using ordinal or nominal regression, the proportional odds assumption was assessed. If the assumption was violated, the mediator variable was treated as nominal and, if the assumption was not violated, it was treated as ordinal.

In the final step, we regressed the outcomes (Y) onto ASES (X) and lifestyle factors (M), covariates (Co) and interaction terms of exposure (X.Mod) and mediators (M.Mod) by age (the moderator), if it was identified in step 2. The four steps are described by the following regression equations:

Step 1 effect of ASES on outcome (b_1_)

Y = b_1_(X) + b_2_(Mod) + b_3_(Co)

Step 2 moderators of effect of ASES on outcome (b_6_)

Y = b_4_(X) + b_5_(Mod) + b_6_(X.Mod) + b_7_(Co)

Step 3 effect of ASES (b_8_) and its moderators (b_10_) on lifestyle factors (mediator)

M = b_8_(X) + b_9_(Mod) + b_10_(X.Mod)* + b_11_(Co)

Step 4 exposure-adjusted effect of mediator (b_13_) and its moderators (b_16_) on outcome, and direct effect of ASES (b_12_) and its moderator (b_15_) on outcome

Y = b_12_(X) + b_13_(M) + b_14_(Mod) + b_15_(X.Mod)* + b_16_(M.Mod)* + b_17_(Co)

where b_1_, ….,b_17_ are regression coefficients and the interaction terms denoted by * are included when b_6_ is statistically significant in step 2. Mediation and mediated moderation were confirmed using the joint-significance test. According to this test, mediation is confirmed if both b_8_ and b_13_ regression coefficients are statistically significant. Mediated moderation is confirmed when both b_10_ and b_13_ regression coefficients are statistically significant and/or both b_8_ and b_16_ are statistically significant (Cerin et al., 2018; Muller et al., 2005). We remark that some mathematical details have been omitted in the equations in step 1–4 (e.g., the equations for nominal and ordinal regression are not correctly represented from a mathematical standpoint here), as the purpose was to highlight how mediation and mediated moderation are identified in a non-technical way.

The mediation analysis was conducted in R V. 4.0.4 using the packages ‘lme4’, ‘mgcv’ and ‘medflex’, all other analyses were performed using STATA V.16. Medflex package from R was used to calculate combined mediation effect of all mediators together and the proportion of the mediated effect (PME) (Steen et al., 2017). PME is the proportion of the total effect mediated through a mediator. Here, we also note that our use of the term “effect” is purely statistical and does not imply causality.

## 3 Results

Table 1A, Table 1BA and 1B report the age-adjusted baseline characteristics of women and men by ASES quartile, from lowest to highest level of ASES. The mean age of the participants was 57 years. Those living in areas with a lower ASES score had higher BMI, waist circumference, and total/HDL cholesterol ratio, and had lower fruit- and vegetable intake, were more likely to be smokers, be a non-drinker or have the highest alcohol intake per drinking session, be physically inactive, and had a higher prevalence of hypertension and diabetes (statistically significant in women only) compared to those with a higher ASES score.

**Table 1A:** Age-adjusted baseline characteristics of women by area-level socio-economic status (ASES) score in quartiles. The Tromsø Study 2015–2016.

Baseline characteristics	ASES score in quartiles				P-value for equality between ASES
	Lowest (n = 2554)	Second (n = 2609)	Third (n = 2522)	Highest (n = 2417)	
Age, years	58.0 (11.2)	56.2 (10.8)	55.9 (11.0)	55.7 (10.6)	<0.0001
Body mass index, kg/m^2^	27.6 (5.1)	27.1 (4.9)	26.5 (4.8)	26.1 (4.6)	<0.0001
Waist circumference, cm	92.6 (13.0)	91.1 (12.9)	89.8 (12.7)	88.8 (12.3)	<0.0001
Total cholesterol, mmol/l	5.56 (1.08)	5.56 (1.05)	5.52 (1.04)	5.54 (1.06)	0.48
HDL-cholesterol, mmol/l	1.70 (0.50)	1.72 (0.47)	1.75 (0.49)	1.79 (0.49)	<0.0001
Total/HDL cholesterol ratio	3.53 (1.18)	3.45 (1.13)	3.38 (1.17)	3.31 (1.10)	<0.0001
Alcohol (per drinking session), %					<0.0001
No alcohol	8.0	6.5	5.8	3.7	
1–2 units	64.1	66.1	70.2	70.8	
3–4 units	22.9	23.8	20.8	22.2	
5 or more units	5.0	3.6	3.1	3.3	
Smoking, %					<0.0001
No Smoking	33.4	34.5	39.1	40.6	
Previous smoking	45.4	46.7	44.2	45.8	
Occasional smoking	3.4	3.5	3.3	4.3	
Daily smoking	17.7	15.2	13.4	9.3	
Snuff use, %					0.070
No Snuff user	94.1	93.8	94.4	93.5	
Previous snuff user	2.4	2.3	2.8	3.2	
Occasional snuff user	0.8	0.5	0.5	0.4	
Daily snuff user	2.7	3.4	2.4	2.9	
Physical activity, %					<0.0001
Sedentary	15.4	14.4	12.4	11.1	
Light activity	66.9	65.8	64.4	63.8	
Moderate-to-vigorous activity	17.7	19.7	23.2	25.1	
Hypertension, %	37.7	34.3	34.3	31.5	<0.0001
Diabetes, %	5.2	4.1	3.1	3.1	0.0002
**Foods/nutrients (subgroup)**	**Lowest (n=1552)**	**Second (n=1353)**	**Third (n=1449)**	**Highest (n=1354)**	
Fruits and vegetables, g/day	591 (343)	608 (333)	599 (337)	624 (323)	0.053
Saturated fat, g/day	31 (13)	31 (13)	30 (11)	31 (12)	0.058
Sugar, g/day	30 (29)	28 (28)	28 (28)	28 (28)	0.21

*Area level socio-economic status is divided into quartiles from lowest to highest level; ASES-Area level socio-economic status; p-value for linear trend.

**Table 1B:** Age-adjusted baseline characteristics of men by area-level socio-economic status (ASES) score in quartiles. The Tromsø Study 2015–16.

Baseline characteristics	ASES score in quartiles				P-value for equality between ASES
	Lowest (n = 2435)	Second (n = 2376)	Third (n = 2448)	Highest (n = 2054)	
Age, years	58.2 (11.1)	57.5 (11.4)	56.3 (11.1)	56.2 (10.8)	<0.0001
Body mass index, kg/m2	28.3 (4.1)	27.8 (4.0)	27.7 (3.9)	27.3 (3.9)	<0.0001
Waist circumference, cm	101.6 (11.4)	100.2 (11.1)	99.5 (10.9)	98.9 (11.2)	<0.0001
Total cholesterol, mmol/l	5.40 (1.10)	5.37 (1.07)	5.39 (1.04)	5.42 (1.04)	0.46
HDL-cholesterol, mmol/l	1.36 (0.40)	1.38 (0.40)	1.41 (0.40)	1.43 (0.41)	<0.0001
Total/HDL cholesterol ratio	4.26 (1.42)	4.18 (1.38)	4.11 (1.39)	4.07 (1.33)	<0.0001
Alcohol (per drinking session), %					<0.0001
No alcohol	4.0	3.8	2.9	2.2	
1–2 units	47.7	52.3	55.6	55.2	
3–4 units	31.0	30.3	31.3	31.2	
5 or more units	17.3	13.6	10.3	11.4	
Smoking, %					<0.0001
No Smoking	31.9	36.6	41.4	41.9	
Previous smoking	45.6	45.0	44.7	43.6	
Occasional smoking	5.4	5.1	4.5	5.5	
Daily smoking	17.1	13.2	9.4	9.0	
Snuff use, %					0.64
No Snuff user	77.5	75.4	76.4	75.6	
Previous snuff user	9.8	10.7	10.5	11.3	
Occasional snuff user	1.2	1.2	1.5	1.5	
Daily snuff user	11.6	12.6	11.5	11.6	
Physical activity, %					<0.0001
Sedentary	19.9	15.1	13.4	11.6	
Light activity	51.6	50.3	49.4	50.5	
Moderate-to-vigorous activity	28.5	34.6	37.2	37.9	
Hypertension, %	46.8	47.1	43.7	43.6	0.009
Diabetes, %	6.2	5.7	4.9	4.9	0.13
**Foods/nutrients (subgroup)**	**Lowest (n=1353)**	**Second (n=1352)**	**Third (n=1074)**	**Highest (n=1234)**	
Fruits and vegetables, g/day	495 (317)	503 (309)	552 (380)	543 (315)	<0.0001
Saturated fat, g/day	37 (15)	36 (13)	35 (13)	36 (13)	0.055
Sugar, g/day	34 (30)	34 (32)	33 (31)	33 (29)	0.73

*Area level socio-economic status is divided into quartiles from lowest to highest level; ASES-Area level socio-economic status; p-value for linear trend.

### 3.1 Associations between area level socio-economic status (exposure) and lifestyle factors (mediators) and age as moderator

ASES was significantly associated with lifestyle factors such as smoking, alcohol use, physical activity level and food and nutrient intake (fruits/vegetables and fat intake only men) in both sexes. We found that ASES was inversely associated with the odds of being a current or previous smoker versus being a non-smoker in both sexes (Table 2). These associations were moderated by age, showing stronger effects in older participants. For alcohol consumption, we found that higher ASES was associated with higher odds of consuming 1–4 units of alcohol per drinking session versus consuming no alcohol in both sexes (Table 2). ASES was not significantly associated with the odds of consuming five or more units of alcohol versus consuming no alcohol (except for younger women). Age moderated the associations of ASES and alcohol consumption in women only, where stronger associations were observed in younger women. ASES was also positively associated with physical activity in both sexes, with stronger effects being observed in older participants (Table 2). There was no significant association of ASES with snuff use.

**Table 2:** Associations between area-level socio-economic status (exposure) and lifestyle behaviors (mediators) by sex and as main effects or effects moderated by age (n = 19415). The Tromsø Study 2015–2016.

Mediators	Main effects not moderated by age		Effects moderated by age					
	Women	Men	Women			Men		
	OR (95% CI)	OR (95% CI)	OR (95% CI)			OR (95% CI)		
	**-**	**-**	−1 SD	Mean age	+1 SD	−1 SD	Mean age	+1 SD
Smoking (ref: Never)								
Previous^a^			0.98 (0.89, 1.08)	**0.86 (0.78, 0.92)**	**0.75 (0.68, 0.83)**	**0.81 (0.73, 0.90)**	**0.84 (0.78, 0.91)**	**0.88 (0.79, 0.98)**
Occasional^a^			**1.45 (1.05, 2.00)**	1.04 (0.85, 1.28)	**0.75 (0.60, 0.95)**	1.10 (0.86, 1.42)	0.91 (0.78, 1.06)	**0.75 (0.62, 0.90)**
Current^a^			**0.72 (0.61, 0.85)**	**0.59 (0.52, 0.67)**	**0.48 (0.41, 0.57)**	**0.65 (0.55, 0.77)**	**0.56 (0.49, 0.64)**	**0.48 (0.41, 0.57)**
Alcohol (ref: 0 units)	–					No significant interaction with age	**-**	**-**
1–2 units^a^		**1.59 (1.34, 1.89)**	**1.75 (1.51, 2.03)**	**1.58 (1.36, 1.84)**	**1.43 (1.13, 1.81)**			
3–4 units^a^		**1.51 (1.27, 1.79)**	**1.86 (1.54, 2.25)**	**1.45 (1.22, 1.72)**	1.13 (0.88, 1.46)			
5 or more^a^		1.10 (0.90, 1.35)	**1.91 (1.24, 2.92)**	1.25 (0.96, 1.63)	0.82 (0.61, 1.11)			
Snuff (ref: Never)		1.05 (0.96, 1.15)^a^	No significant interaction with age	–	–	No significant interaction with age	–	–
Previous^a^	**1.24 (1.02, 1.51)**							
Occasional^a^	0.69 (0.45, 1.06)							
Current^a^	0.96 (0.78, 1.18)							
Physical activity (ref: Sedentary)	**-**	**-**	**1.13 (1.02, 1.26)**^**a**^	**1.32 (1.21, 1.45)**^**a**^	**1.54 (1.38, 1.72)**^**a**^			
Light activity^a^						**1.19 (1.06, 1.34)**	**1.32 (1.21, 1.44)**	**1.47 (1.29, 1.66)**
Moderate-to-vigorous activity^a^						**1.25 (1.07, 1.47)**	**1.57 (1.38, 1.78)**	**1.97 (1.68, 2.31)**
**Dietary variables (women n= 5708, men n=5013)**								
	**B (95%CI)**	**B (95%CI)**	**B (95%CI)**			**B (95%CI)**		
Fruits and vegetables (g/day)	**21.65 (8.38, 34.91)**	**29.93 (16.49, 43.07)**	No significant interaction with age	**-**	**-**	No significant interaction with age	**-**	**-**
Saturated fat (g/day)	−0.03 (−0.59, 0.53)	**−0.79 (-1.38, -0.21)**	No significant interaction with age	**-**	**-**	No significant interaction with age	**-**	**-**
Sugar (g/day)	−0.90 (−2.11, 0.33)	−0.99 (−2.17, 0.20)	No significant interaction with age	**-**	**-**	No significant interaction with age	**-**	**-**

All models are adjusted for age and individual-level socio-economic status and accounted for area-level clustering; All effects are estimated per unit increase in ASES; OR = odds ratio; CI = confidence interval; SD = standard deviation; B = regression coefficient; ref = reference category; proportional odds assumption was tested to see if the variable is nominal or ordinal; a = Treated as an ordinal variable (therefore only one value).

a Nominal mediator, using generalized additive mixed models with multinominal variance.

ASES was positively associated with fruits and vegetables intake in both sexes and negatively associated with fat intake in men.

### 3.2 Associations between area level socio-economic status (exposure) and CVD risk factors and age as moderator

ASES was significantly associated with all the outcome variables (Total effect; Table 3A, Table 3B, Table 3CA-3C). The associations were inverse, that is, higher levels of ASES were associated with a decrease in CVD risk factors. Stronger associations were found in women compared to men in several outcome variables. A unit increase in ASES was associated with 2.3 cm (95% CI -2.93, −1.66) decrease in waist circumference in women (of average age) and 1.4 cm (95% CI -1.85, −0.93) in men (Table 3A). Similarly, the odds of diabetes decreased by 32% (95% CI 0.58, 0.81) per unit increase in ASES in women and by 17% (95% CI 0.73, 0.95) with each unit increase in ASES among men (Table 3B). Age was a significant moderator of the association between ASES with total/HDL cholesterol ratio in both sexes and waist circumference in women (Table 3A, Table 3B, Table 3CA-3C), where the effects of ASES were stronger in older participants. Adding random slopes for age to the model did not change the results except for BMI and waist circumference in men. Thus, random slopes for age were added to those models.

**Table 3A:** Results of mediation analysis for women: total, direct, and indirect effects of area-level socio-economic status on cardiovascular risk factors (outcomes) (n = 10102). The Tromsø Study 2015–2016.

Effects	BMI	Total/HDL Cholesterol ratio	Waist circumference	Hypertension	Diabetes
	B (95% CI)	B (95% CI)	B (95% CI)	OR (95% CI)	OR (95% CI)
Total effect^a^	**−0.94 (-1.09, -0.79)** No significant interaction with age			**0.79 (0.74, 0.86)** No significant interaction with age	**0.68 (0.58, 0.81)** No significant interaction with age
Association at:^e^					
−1 SD		**−0.10 (-0.16, -0.05)**	**−1.84 (-2.57, -1.11)**		
Mean age		**−0.14 (-0.18, -0.10)**	**−2.30 (-2.93, -1.66)**		
+1 SD		**−0.18 (-0.24, -0.13)**	**−2.76 (-3.51, -2.01)**		
Direct effect^b^	**−0.83 (-0.99, -0.68)** No significant interaction with age	**−0.10 (-0.13, -0.07)** No significant interaction with age	**−1.95 (-2.35, -1.58)** No significant interaction with age	**0.80 (0.75, 0.88)** No significant interaction with age	**0.70 (0.59, 0.83)** No significant interaction with age
Association at: e					
−1 SD					
Mean age					
+1 SD					
Indirect effect, combined^c^	**−0.11(-0.17, -0.04)**	**−0.05 (-0.06, -0.03)**	**−0.39 (-0.54, -0.22)**	0.99 (0.96, 1.00)	0.97 (0.92, 1.03)
PME^d^	0.12 (0.05, 0.19)	0.32 (0.23, 0.44)	0.17 (0.10, 0.24)	0.05 (−0.06, 0.15)	0.07 (−0.06, 0.23)
Exposure-adjusted effects of mediators:					
Smoking (ref: Never)	Mediation **0.47 (0.26, 0.68)** −0.11 (−0.63, 0.40) **−0.92 (-1.22, -0.61)**	Mediated moderation	Mediated moderation	Mediation 0.94 (0.85, 1.05) **0.63 (0.45, 0.84)** **0.76 (0.65, 0.88)**	Mediation **0.77 (0.62, 0.97)** 0.76 (0.39, 1.46) 0.74 (0.53, 1.02)
Previous					
Association at: e					
−1 SD		−0.0002 (−0.07, 0.07)	**1.67 (0.91, 2.41)**		
Mean age		**0.06 (0.01, 0.11)**	**1.69 (1.14, 2.23)**		
+1 SD		**0.12 (0.05, 0.19)**	**1.71 (0.95, 2.47)**		
Occasional					
Association at: e					
−1 SD		0.02 (−0.21, 0.25)	2.07 (−0.48, 4.61)		
Mean age		−0.007 (−0.14, 0.12)	1.26 (−0.21, 2.72)		
+1 SD		0.04 (−0.19, 0.11)	0.45 (−1.23, 2.12)		
Current					
Association at: e					
−1 SD		**0.18 (0.07, 0.29)**	**−2.87 (-4.05, -1.69)**		
Mean age		**0.40 (0.33, 0.48)**	−0.65 (−1.44, 0.13)		
+1 SD		**0.63 (0.05, 0.19)**	**1.57 (0.43, 2.70)**		
Alcohol (ref: 0 units)	Mediation	Mediated moderation	Mediated moderation	No mediation	Mediation
1–2 units					
Association at: e					
−1 SD					
Mean age	**−0.48 (-0.89, -0.08)**	**−0.16 (-0.26, -0.05)**	−1.08 (−2.24, 0.07)	0.90 (0.73, 1.09)	**0.57 (0.42, 0.78)**
+1 SD					
3–4 units					
Association at: e					
−1 SD	0.21 (−0.23, 0.65)	**−0.13 (-0.24, -0.02)**	0.99 (−0.25, 2.23)	1.02 (0.82, 1.27)	**0.52 (0.35, 0.77)**
Mean age					
+1 SD					
5 or more					
Association at: e	**1.32 (0.68, 1.94)**	0.04 (−0.16, 0.23)	**2.18 (0.04, 4.31)**	1.15 (0.84, 1.58)	**0.29 (0.12, 0.69)**
−1 SD					
Mean age					
+1 SD					
Snuff (ref: Never)	No mediation	No mediation	No mediation	No mediation	No mediation
Previous	0.07 (−0.52, 0.67)	−0.09 (−0.26, 0.07)	0.47 (−1.41, 2.34)	0.88 (0.64, 1.22)	1.11 (0.54, 2.28)
Occasional	0.07 (−1.24, 1.39)	−0.09 (−0.55, 0.38)	−2.35 (−7.46, 2.79)	0.74 (0.33, 1.67)	0.73 (0.10, 5.33)
Current	−0.37 (−0.94, 0.20)	−0.06 (−0.28, 0.17)	−0.39 (−2.85, 2.07)	0.87 (0.62, 1.21)	0.40 (0.13, 1.25)
Physical activity (ref: Sedentary) Light activity Association at:^e^ −1 SD Mean age +1 SD Moderate-to-vigorous activity Association at:^e^ −1 SD Mean age +1 SD	Mediation **−2.17 (-2.45, -1.89)** **−3.41 (-3.74, -3.09)**	Mediated moderation	Mediated moderation	Mediation **0.71 (0.62, 0.81)** **0.53 (0.45, 0.63)**	Mediation **0.50 (0.39, 0.63)** **0.37 (0.26, 0.53)**
		**−0.24 (-0.32, -0.15)**	**−6.16 (-7.10, -5.23)**		
		**−0.31 (-0.37, -0.24)**	**−5.69 (-6.42, -4.97)**		
		**−0.38 (-0.47, -0.28)**	**−5.22 (-6.24, -4.20)**		
		**−0.38 (-0.49, -0.28)**	**−8.32 (-9.51, -7.14)**		
		**−0.58 (-0.66, -0.50)**	**−9.25 (-10.10, -8.39)**		
		**−0.78 (-0.88, -0.67)**	**−10.2 (-11.32, -9.03)**		

BMI = body mass index; CI = confidence interval; SD = standard deviation; B = regression coefficient; OR = odds ratio; PME = proportion of mediated effect.

a Association between exposure and outcome: main effect presented when age is not a significant moderator; age-specific associations presented when age was a significant moderator.

b Association between exposure and outcome not explained by mediators.

c Association between exposure and outcome through mediators (dietary variables not included).

d The proportion of the total effect mediated by mediator(s). All models were adjusted for age and individual-level SES and accounted for area-level clustering.

e If significant interaction with moderator (age).

**Table 3B:** Results of mediation analysis for men: total, direct, and indirect effects of area-level socio-economic status on cardiovascular risk factors (outcomes) (n = 9313). The Tromsø Study 2015–2016.

Effects	BMI	Total/HDL Cholesterol ratio	Waist circumference	Hypertension	Diabetes
	B (95% CI)	B (95% CI)	B (95% CI)	OR (95% CI)	OR (95% CI)
Total effect^a^	**−0.54 (-0.66, -0.41)** No significant interaction with age	−0.02 (−0.07, 0.04) **−0.10 (-0.14, -0.06)** **−0.19 (-0.25, -0.13)**	**−1.46 (-1.79, -1.13)** No significant interaction with age	**0.91 (0.85, 0.97)** No significant interaction with age	**0.83 (0.73, 0.95)** No significant interaction with age
Association at:*					
−1 SD					
Mean age					
+1 SD					
Direct effect^b^	**−0.42 (-0.53, -0.28)** No significant interaction with age	0.005 (−0.05, 0.06) **−0.06 (-0.10, -0.02)** **−0.1 (-0.18, -0.06)**	**−0.95 (-1.23, -0.57)** No significant interaction with age	**0.92 (0.86, 0.98)** No significant interaction with age	**0.86 (0.75, 0.99)** No significant interaction with age
Association at:*					
−1 SD					
Mean age					
+1 SD					
Indirect effect, combined^c^	**−0.12 (-0.17, -0.08)**	**−0.04 (-0.05, -0.03)**	**−0.51 (-0.70, -0.42)**	0.98 (0.97, 1.00)	**0.96 (0.93, 0.99)**
PME^d^	0.22 (0.13, 0.32)	0.43 (0.27, 0.72)	0.35 (0.25, 0.48)	0.18 (0.01, 0.58)	0.23 (0.03, 0.84)
Exposure-adjusted effects of mediators:					
Smoking (ref: Never)	Mediation **0.60 (0.42, 0.78)** **0.49 (0.11, 0.87)** **−0.58 (-0.85, -0.31)**	Mediated moderation −0.02 (−0.10, 0.07) **0.06 (0.00005, 0.12)** **0.14 (0.05, 0.23)** **0.33 (0.09, 0.57)** **0.34 (0.21, 0.48)** **0.36 (0.20, 0.52)** 0.14 (−0.01, 0.28) **0.34 (0.24. 0.43)** **0.53 (0.40, 0.67)**	Mediation **2.64 (2.13, 3.14)** **2.52 (1.46, 3.57)** 0.29 (−0.46, 1.05)	Mediation **1.21 (1.09, 1.33)** 0.94 (0.77, 1.16) 0.87 (0.75, 1.00)	Mediation **1.27 (1.03, 1.57)** 0.72 (0.41, 1.26) 0.97 (0.70, 1.35)
Previous					
Association at:*					
−1 SD					
Mean age					
+1 SD					
Occasional					
Association at:*					
−1 SD					
Mean age					
+1 SD					
Current					
Association at:*					
−1 SD					
Mean age					
+1 SD					
Alcohol (ref: 0 units)	Mediation −0.10 (−0.47, 0.44) **0.83 (0.36, 1.29)** **1.47 (0.97, 1.96)**	No mediation −0.13 (−0.29, 0.04) −0.08 (−0.25, 0.09) 0.14 (−0.04, 0.32)	Mediation −0.24 (−1.52, 1.04) **2.23 (0.92, 3.54)** **4.44 (3.04, 5.84)**	Mediation 1.05 (0.81, 1.36) **1.34 (1.03, 1.74)** **1.51 (1.14, 1.99)**	Mediation **0.63 (0.42, 0.93)** **0.65 (0.43, 0.98)** 0.83 (0.52, 1.31)
1–2 units					
Association at:*					
−1 SD					
Mean age					
+1 SD					
3–4 units					
Association at:*					
−1 SD					
Mean age					
+1 SD					
5 or more					
Association at:*					
−1 SD					
Mean age					
+1 SD					
Snuff (ref: Never)	No mediation	No mediation	No mediation	No mediation	No mediation
Previous	**0.30 (0.03, 0.57)**	0.08 (−0.02, 0.19)	**1.36 (0.61, 2.12)**	1.15 (0.99, 1.33)	0.93 (0.65, 1.32)
Occasional	−0.06 (−0.76, 0.64)	−0.06 (−0.43, 0.30)	0.45 (−1.51, 2.40)	0.72 (0.47, 1.10)	**2.17 (1.08, 4.37)**
Current	0.21 (−0.04, 0.47)	0.01 (−0.09, 0.12)	**0.96 (0.23, 1.69)**	**1.21 (1.05, 1.39)**	0.91 (0.64, 1.28)
Physical activity (ref: Sedentary)	Mediation **−1.41 (-1.64, -1.18)** **−2.30 (-2.54, -2.05)**	Mediated moderation **−0.17 (-0.28, -0.07)** **−0.18 (-0.26, -0.10)** **−0.19 (-0.31, -0.08)** **−0.35 (-0.47, -0.23)** **−0.54 (-0.63, -0.46)** **−0.73 (-0.85, -0.62)**	Mediation **−4.91 (-5.55, -4.27)** **−8.59 (-9.27, -7.90)**	Mediation **0.84 (0.74, 0.96)** **0.66 (0.58, 0.76)**	Mediation **0.59 (0.47, 0.73)** **0.40 (0.31, 0.53)**
Light activity					
Association at:*					
−1 SD					
Mean age					
+1 SD					
Moderate-to-vigorous activity					
Association at:*					
−1 SD					
Mean age					
+1 SD					

BMI = body mass index; CI = confidence interval; SD = standard deviation; B = regression coefficient; OR = odds ratio; PME = proportion of mediated effect.

a Association between exposure and outcome: main effect presented when age is not a significant moderator; age-specific associations presented when age was a significant moderator.

b Association between exposure and outcome not explained by mediators.

c Association between exposure and outcome through mediators (dietary variables not included).

d The proportion of the total effect mediated by mediator(s). All models were adjusted for age and individual-level SES and accounted for area-level clustering and random slope for age where applicable. *if significant interaction with moderator (age).

**Table 3C:** Results of mediation analysis for women and men in sub analysis: total, direct, and indirect effects of area-level socio-economic status on cardiovascular risk factors (outcomes) (n = 10721). The Tromsø Study 2015–2016.

Effects	BMI	Total/HDL Cholesterol ratio	Waist circumference	Hypertension	Diabetes
**Women (n= 5708)**	B (95% CI)	B (95% CI)	B (95% CI)	OR (95% CI)	OR (95% CI)
Total effect^a^	**−0.86 (-1.14, -0.58)** No significant interaction with age	**−0.15 (-0.20, -0.09)** No significant interaction with age	**−2.04 (-2.81, -1.27)** No significant interaction with age	**0.82 (0.74, 0.91)** No significant interaction with age	**0.72 (0.58, 0.89)** No significant interaction with age
Association at:^e^					
−1 SD					
Mean age					
+1 SD					
Direct effect^b^	**−0.74 (-0.95, -0.55)** No significant interaction with age	**−0.09 (-0.14, -0.05)** No significant interaction with age	**−1.62 (-2.18, -1.13)** No significant interaction with age	**0.83 (0.75, 0.92)** No significant interaction with age	**0.74 (0.59, 0.94)** No significant interaction with age
Association at: e					
−1 SD					
Mean age					
+1 SD					
Indirect effect, combined^c^ PME^d^	**−0.16 (-0.23, -0.07)** 0.18 (0.09, 0.28)	**−0.05 (-0.06, -0.03)** 0.34 (0.22, 0.51)	**−0.49 (-0.68, -0.26)** 0.23 (0.13, 0.36)	0.98 (0.95, 1.01) 0.09 (−0.06, 0.28)	0.96 (0.90, 1.02) 0.12 (−0.08, 0.48)
Exposure-adjusted effects of mediators:					
Fruits and vegetables (g/day)	−0.006 (−0.13, 0.12) No mediation	−0.008 (−0.04, 0.02) No mediation	−0.20 (−0.5, 0.13) No mediation	0.99 (0.94, 1.06) No mediation	1.03 (0.90, 1.18) No mediation
Saturated fat (g/day)	−0.02 (−0.15, 0.10) No mediation	0.008 (−0.02, 0.04) No mediation	−0.03 (−0.35, 0.298) No mediation	**0.91 (0.85, 0.97)** No mediation	0.97 (0.84, 1.11) No mediation
Sugar (g/day)	0.06 (−0.06, 0.19) No mediation	0.03 (−0.003, 0.06) No mediation	0.11 (−0.21, 0.44) No mediation	**0.92 (0.86, 0.99)** No mediation	**0.78 (0.64, 0.94)** No mediation
**Men (n=5013)**	B (95% CI)	B (95% CI)	B (95% CI)	OR (95% CI)	OR (95% CI)
Total effect^a^	**−0.64 (-0.79, -0.49)** No significant interaction with age			**0.88 (0.81, 0.97)** No significant interaction with age	**0.81 (0.68, 0.97)** No significant interaction with age
Association at: e					
−1 SD		−0.008 (−0.08, 0.07)	**−1.08 (-1.67, -0.50)**		
Mean age		**−0.11 (-0.17, -0.05)**	**−1.78 (-2.20, -1.36)**		
+1 SD		**−0.21 (-0.29, -0.13)**	**−2.48 (-3.09, -1.87)**		
Direct effect^b^	**−0.56 (-0.71, -0.41)** No significant interaction with age			**0.89 (0.81, 0.96)** No significant interaction with age	**0.84 (0.69, 0.99)** No significant interaction with age
Association at: e					
−1 SD		0.01 (−0.06, 0.09)	**−0.88 (-1.43, -0.33)**		
Mean age		**−0.07 (-0.12, -0.01)**	**−1.36 (-1.76, -0.96)**		
+1 SD		**−0.14 (-0.22, -0.07)**	**−1.84 (-2.43, -1.26)**		
Indirect effect, combined^c^ PME^d^	**−0.08 (-0.13, -0.01)** 0.13 (0.04, 0.22)	**−0.04 (-0.06, -0.02)** 0.38 (0.20, 0.79)	**−0.41 (-0.55, -0.20)** 0.23 (0.13, 0.34)	0.99 (0.98, 1.03) 0.03 (−0.20, 0.30)	0.96 (0.92, 1.03) 0.18 (−0.11, 0.89)
Exposure-adjusted effects of mediators:					
Fruits and vegetables (g/day)	0.004 (−0.10, 0.11) No mediation	**−0.04 (-0.08, -0.01)** Mediation	−0.15 (−0.46;0.16) No mediation	**0.94 (0.88, 0.99)** Mediation	1.01 (0.89, 1.14) No mediation
Saturated fat (g/day)	**−0.24 (-0.35, -0.13)** Mediation	−0.01 (−0.05;0.02) No mediation	**−0.45 (-0.76;-0.14)** Mediation	**0.85 (0.80, 0.91)** Mediation	1.06 (0.94, 1.20) No mediation
Sugar (g/day)	**−0.19 (-0.30, -0.08)** No mediation	0.03 (−0.01, 0.07) No mediation		**0.91 (0.85, 0.96)** No mediation	**0.70 (0.59, 0.82)** No mediation
Association at: e			No mediation		
−1 SD			**−1.19 (-1.65, -0.74)**		
Mean age			**−0.50 (-0.81, -0.19)**		
+1 SD			0.19 (−0.21, 0.59)		

BMI = body mass index; CI = confidence interval; SD = standard deviation; B = regression coefficient; OR = odds ratio; PME = proportion of mediated effect.

a Association between exposure and outcome: main effect presented when age is not a significant moderator; age-specific associations presented when age was a significant moderator.

b Association between exposure and outcome not explained by mediators.

c Association between exposure and outcome through mediators (smoking, alcohol, snuff and physical activity included).

d The proportion of the total effect mediated by mediator(s). All models were adjusted for age and individual-level SES and accounted for area-level clustering.

e If significant interaction with moderator (age).

### 3.3 Mediators and mediated moderation of associations between area level socio-economic status and CVD risk factors

Lifestyle factors that mediated the associations between ASES and CVD risk factors were smoking, alcohol use and physical activity level (Table 3A, Table 3B, Table 3CA-3C). Smoking and physical activity mediated the association between ASES and BMI, total/HDL cholesterol ratio, waist circumference, hypertension, and diabetes in both sexes. The associations between ASES and total/HDL cholesterol ratio and waist circumference (women) were moderated by age, and the moderating effects were mediated by smoking and physical activity in both sexes (Table 3A, Table 3BA and 3B). Alcohol use mediated the effect of ASES for most of the outcomes. However, alcohol use did not mediate the association between ASES and total/HDL cholesterol ratio (men) and hypertension (women) (Table 3A, Table 3BA and 3B). Snuff use was not a mediator of the associations between ASES and CVD risk factors in both sexes (Table 3A, Table 3BA and 3B) as it was not associated with ASES (Table 2). The total mediated (indirect) effect of ASES was not significant for hypertension for both sexes and diabetes for women (Indirect effect; Table 3A, Table 3BA and 3B). The largest total indirect effect of ASES (through all mediators together) was found in relation to total/HDL cholesterol ratio, with the mediators accounting for 43% of the observed effects (PME; Table 3A, Table 3BA and 3B).

In the subgroup analysis, including the dietary variables, ASES had the largest total indirect effect on total/HDL cholesterol, with the mediators accounting for 34% of the observed effects (PME; Table 3C).

## 4 Discussion

In this cross-sectional population-based study, we found that higher ASES was associated with lower CVD risk independent of individual-level SES. All the associations were mediated by lifestyle factors such as smoking and physical activity in both sexes as well as alcohol use and dietary variables (in men). In Tromsø, areas with high and low SES are located very close to each other but there were large differences between the living areas. We did not find any similar studies performed within areas with such a small geographical proximity. To our knowledge, no previous study has examined the associations between ASES with a wide range of metabolic CVD risk factors independent of individual-level SES and investigated the mediating role of lifestyle factors in these associations by age and sex groups. Number of immigrants in an area might influence the health status of that area. Considering this possibility, we performed sensitivity analyses on one of the outcomes (BMI) and found that the main results were unchanged after including this variable. The results are presented in the supplementary material (page 3–4).

### 4.1 Area-level socioeconomic status and metabolic CVD risk factors

We found that people living in higher ASES had lower metabolic CVD risk factors, such as lower odds of hypertension, diabetes, lower total/HDL cholesterol ratio, BMI, and waist circumference even after adjustment for individual-level SES. Previous studies have shown a well-established association between individual-level SES and cardiovascular health (Beauchamp et al., 2010; Eggen et al., 2014; Ernstsen et al., 2012; Lindroth et al., 2014; Rosengren et al., 2019). However, the independent effects of ASES have been examined to a lesser extent. This study suggests that ASES is an important factor having potential impact on CVD in addition to individual-level SES. ASES is thought to independently contribute to health outcomes due to area-level differences in several characteristics. Social norms related to health behaviors and social interactive mechanism are one of these characteristics (Diez Roux et al., 2001; Galster, 2012). Other underlying pathways might be that persons living in low SES environments are frequently exposed to stressors, and due to limited resources, they also have an increased sensitivity to stressors that are ultimately linked to CVD risk (Diez Roux et al., 2001; Matthews & Gallo, 2011). As both individual-level SES and ASES are shown to contribute independently to health outcomes, health inequalities cannot be established in studies using only one of these SES indicators (Anderson et al., 1997; Haan et al., 1987; Smith et al., 1998; Waitzman & Smith, 1998; Yen & Kaplan, 1999). The role of ASES as a health risk factor needs more attention (Yen & Kaplan, 1999). Other studies reported effects similar to ours independent of individual-level SES. A prospective cohort study in four US communities showed that people living in disadvantaged neighborhoods had higher risk of coronary heart disease (Diez Roux et al., 2001). Prospective population studies in Scotland showed ASES to be inversely associated with CVD risk factors (Hart et al., 1997; Smith et al., 1998). Another longitudinal health survey in the West of Scotland showed inverse associations between neighborhood deprivation and CVD risk factors, such as BMI and waist circumference (Ellaway et al., 1997).

In our study, we also examined whether the associations between ASES and risk of CVD were moderated by age and stratified all analyses by sex. Age was a significant moderator of the association of ASES with cholesterol in both sexes, and with waist circumference in women. All these associations of ASES with CVD risk factors were stronger in older participants. A retrospective study performed in Japan found that waist circumference increased over time regardless of body weight, and the increase was larger in older women compared to men (Ono et al., 2022). The accumulated effect of unhealthy behaviors over the life course could have been more prominent in older compared to younger segments of the population. Since the accumulated effect of an unhealthy lifestyle are seen earlier in markers such as waist and cholesterol compared to hypertension (Downer et al., 2014; Lloyd-Jones et al., 2005), it is understandable that age was found to be a moderator of the associations of only a subset of the examined outcomes. The associations between ASES and CVD risk factors were in the same direction (inverse association) for both women and men in our study. However, significantly stronger associations were found in women compared to men. A prospective population study in Scotland also showed higher BMI among women but not in men living in less deprived areas (Smith et al., 1998). In addition, a cross-sectional study in Australia showed a stronger association between ASES and BMI in women than in men (King et al., 2006). This might be explained by the fact that women tend to be more sociable and interact with neighbors more often than men do (Shye et al., 1995). Therefore, they may be more prone to changing their lifestyle as a result of social-interactive mechanisms, including social contagion and collective socialization (Galster, 2012).

Table 1A, Table 1B shows the prevalence of lifestyle factors and cardiometabolic risk factors in our population according to ASES quartiles. These findings can be interpreted in terms of achievement of the World Health Organization (WHO) targets. For example, WHO has set a target of 15% reduction in physical inactivity by 2030. Table 1A, Table 1B shows a relative difference of over 15% in physical inactivity (sedentariness) between the lowest ASES quartile (15.4%) and the third (12.4%) and highest ASES quartiles (11.1%) in women, and between the lowest ASES quartile (19.9%) and all other ASES quartiles in men (15.1%, 13.4% and 11.6%). This indicates that the differences in physical inactivity prevalence between ASES quartiles are larger than the WHO target of 15% relative reduction in physical inactivity and, hence, are of public health significance. Similarly, the WHO 2030 target related to overweight and obesity states that countries should strive to maintain or reduce the current levels of overweight and obesity. Our study indicates that ASES has a meaningful contribution to this health outcome because the mean BMI was lower with increasing ASES quartiles (Table 1A, Table 1B).

### 4.2 Lifestyle factors as mediators

We found associations between ASES and lifestyle factors such as smoking, alcohol, physical activity, and diet, independent of individual-level SES. This can be explained by a neighborhood effect, which is referred to as characteristics and behaviors of individuals living in the same areas influencing residents’ behavior and wellbeing (Durlauf, 2004; Roosa & White, 2014). Several studies also supported 10.13039/100013615ASES effects independent of individual-level SES. In studies performed in the UK, neighborhood SES was shown to be positively associated with smoking status (Duncan et al., 1999; Kleinschmidt et al., 1995) and negatively associated with consumption of fruits and vegetables (Shohaimi et al., 2004). A longitudinal population based cohort study and a cross-sectional study showed that lower ASES was associated with a decline in physical activity (Cerin & Leslie, 2008; Yen & Kaplan, 1998) and identified individual, social and environmental contributors as mediators (Cerin & Leslie, 2008). The effect on patterns of physical activity might be due to neighborhood differences in the physical environment, availability and quality of public spaces and recreational facilities and perceived neighborhood safety (Diez Roux et al., 2001). A previous analysis from the Tromsø Study showed an inverse association between neighborhood physical activity level and BMI (Sari et al., 2021).

We found that the associations between ASES and metabolic CVD risk factors were mediated by lifestyle factors such as smoking and physical activity. Similarly, alcohol use and diet were mediators for most associations. The development and prevalence of lifestyle risk factors can partly be due to neighborhood characteristics. Thus, lifestyle risk factors may be considered as mediators of the effects of the neighborhood environment on CVD (Diez Roux et al., 2001). Several studies have identified lifestyle behaviors as mediators of the association between ASES and CVD. However, these studies only examined CVD incidence and/or mortality, focused on only one lifestyle behavior, or reported findings that were not adjusted for individual-level SES. For example, a large population-based cohort study in the US and UK showed that low ASES was significantly associated with higher risk of mortality and CVD, and these associations were mediated by lifestyle factors such as smoking, alcohol, physical activity, and diet (Zhang et al., 2021). Another prospective study among mid-to-older aged adults from Australia found low ASES to be significantly associated with CVD incidence independent of individual-level SES, and physical activity to be a mediator of this association (Saghapour et al., 2021).

In our study, the association between ASES and lifestyle behaviors were similar in men and women, but stronger effects were seen in older participants. Lifestyle factor are the most important modifiable risk factors contributing to the prevention of NCDs (Zhu et al., 2022). Public health intervention promoting lifestyle behaviors can be tailored to focus on older populations to prevent further degradation of their health. At the same time, the stronger relationship seen in the older population likely reflects the accumulated effects of unhealthy behaviors over the life-course, suggesting that policies and interventions should also target the younger generation. Some examples of initiatives include incentives for non-smokers or for growing vegetables in the garden, public gym facilities to encourage physical activity, and control of alcohol outlets. Furthermore, our finding suggests that public health initiatives should not only target lifestyles at the individual level but also consider neighborhood factors providing more health-related resources and more opportunities to disadvantaged areas.

### 4.3 Strengths and limitations

A major strength of our study was the use of a large sample of both sexes included from a population-based study with high attendance. This study included the use of several validated questionnaires and objective measures using standard methods to measure mediators and outcome variables. The variables constituting the exposure variable ASES were taken from Statistics Norway. Norway has a personal identification number that allows exact matching of population register data, which was used to link data from Statistics Norway and the Tromsø Study.

Possible limitations include the cross-sectional design which limits causal inference. To properly examine causal environmental effects on behaviors and CVDs, we would require sufficient variability in changes of exposures across time. Also, effects of environmental exposures on CVD are likely cumulative and develop across many years. Cross-sectional studies are more likely to capture the cumulative effects of and long-term exposures to neighborhood factors (reflected in inter-area and inter-individual differences) than most longitudinal studies because the latter usually cover a relatively short time period (3–5 years). Changes in behaviors across time could have been examined. However, ASES is quite stable over time and population mobility across areas is not high in Tromsø. In addition, in the areas with greater internal migration, immigrants are from outside the municipality rather than across the municipality areas (Tromsø kommune, 2019). We believe that, in the context of this particular research field, evidence should be based on both cross-sectional and longitudinal (and quasi-experimental) studies, being mindful of the shortcomings of each type. The use of self-reported questionnaires for assessing smoking, alcohol, snuff, physical activity, dietary intake, diabetes, and antihypertensive medication use is another limitation. Certain desired habits tend to be overreported such as physical activity and certain less acceptable habits such as smoking, or alcohol consumption are underreported, which could result in misclassification.

## 5 Conclusion

Living in lower ASES areas is associated with higher CVD risk due to unhealthy lifestyle behaviors including smoking, high alcohol consumption and physical inactivity, independent of individual-level SES in both sexes. This association was stronger in women than in men, and in older than younger participants. Identifying the behavioral pathways in the association between ASES and CVD risk factors can help to develop targeted public health intervention aimed at reducing health inequalities by promoting healthy lifestyle behaviors. In addition, programs that target the demographic profiles of specific communities can be implemented if we understand the role of area of living and the mechanisms through which it affects people's health.

## Funding

The Tromsø Study has been supported by the 10.13039/501100007137Northern Norway Regional Health Authority and the 10.13039/100007465UiT The Arctic University of Norway. Annika Rosengren's research was supported by 10.13039/501100004359Swedish Research Council for research on CVD risk factors (2018–02527). Sweta Tiwari's research was supported by The Tromsø Study and RCN grant ES617148/289440; Healthy choices and the social gradient.

## Ethical statement

The Tromsø Study complies with the declaration of Helsinki and has been approved by the Regional Committee for Medical and Health Research Ethics (REK), the Data Inspectorate, and the Norwegian Directorate of Health. All participants provided written informed consent. The current study was approved by REK North (reference 132624) and evaluated by the Norwegian Centre for Research Data.

## CRediT authorship contribution statement

**Sweta Tiwari:** Methodology, Software, Validation, Formal analysis, Investigation, Data curation, Writing – original draft, Visualization, Project administration, All authors have reviewed and approved the final version of the manuscript. **Ester Cerin:** Conceptualization, Supervision, Methodology, Software, Validation, Writing – review & editing, All authors have reviewed and approved the final version of the manuscript. **Tom Wilsgaard:** Methodology, Software, Validation, Writing – review & editing, All authors have reviewed and approved the final version of the manuscript. **Ola Løvsletten:** Methodology, Software, Validation, Writing – review & editing, All authors have reviewed and approved the final version of the manuscript. **Inger Njølstad:** Funding acquisition, Writing – review & editing, All authors have reviewed and approved the final version of the manuscript. **Sameline Grimsgaard:** Funding acquisition, Writing – review & editing, All authors have reviewed and approved the final version of the manuscript. **Laila A. Hopstock:** Writing – review & editing, All authors have reviewed and approved the final version of the manuscript. **Henrik Schirmer:** Writing – review & editing, All authors have reviewed and approved the final version of the manuscript. **Annika Rosengren:** Writing – review & editing, All authors have reviewed and approved the final version of the manuscript. **Kathrine Kristoffersen:** Writing – review & editing, All authors have reviewed and approved the final version of the manuscript. **Maja-Lisa Løchen:** Conceptualization, Supervision, Writing – review & editing, Project administration, Funding acquisition, All authors have reviewed and approved the final version of the manuscript.

## Declaration of competing interest

None.

## Data Availability

The authors do not have permission to share data.
