# Supplementary material for: Lifestyle factors as mediators of area-level socio-economic differentials in cardiovascular disease risk factors. The Tromsø Study
Source: SSM Popul Health. 2022 Sep 24;19:101241. doi: 10.1016/j.ssmph.2022.101241 (PMC9530956; doi:10.1016/j.ssmph.2022.101241)
Supplement: Multimedia component 1 [file mmc1.docx]

**Supplementary Material**

**Lifestyle factors as mediators of area-level socio-economic differentials in cardiovascular disease risk factors. The Tromsø Study**

1. **Description of study areas**

**Figure 1: Map of Tromsø municipality with areas (Hopstock et al., 2019)**

**Table 1. Total number of individual and mean age according to areas by sex. Tromsø municipality (2015)**

| **Areas** | **Population (N)** | | **Mean age** | |
| --- | --- | --- | --- | --- |
|  | **Women** | **Men** | **Women** | **Men** |
| Bjerkaker-Kveldro | 470 | 404 | 56.0 | 56.5 |
| Dramsveien | 188 | 171 | 56.7 | 57.6 |
| Elverhøy | 278 | 284 | 56.7 | 58.1 |
| Ersfjord-Tromvik-Skulsfjord | 154 | 147 | 56.4 | 57.8 |
| Fagereng | 434 | 324 | 59.2 | 59.6 |
| Finnvik-Kvaløyvågen-øyenen | 153 | 150 | 57.8 | 60.2 |
| Gimle | 143 | 163 | 57.8 | 58.8 |
| Gyllenborg | 347 | 321 | 57.7 | 57.0 |
| Hamna | 358 | 341 | 53.4 | 54.4 |
| Hungeren | 195 | 179 | 56.9 | 55.9 |
| Håkøya-Håkøybotn-Vikran | 221 | 239 | 56.5 | 58.2 |
| Håpet-Langnes | 267 | 167 | 58.2 | 58.6 |
| Kaldfjord-Eidkjosen | 154 | 146 | 54.7 | 57.0 |
| Kaldsletta-Sandvika | 118 | 118 | 53.6 | 54.5 |
| Kroken | 394 | 304 | 57.2 | 57.4 |
| Krokenstranda | 173 | 164 | 54.6 | 55.6 |
| Kvaløysletta | 407 | 435 | 55.5 | 56.8 |
| Lunheim-Tomasjordnes | 373 | 335 | 57.3 | 57.5 |
| Mjelde-Sommarøy-Kattfjord | 187 | 188 | 58.5 | 58.2 |
| Norrøna-Elverhøy | 244 | 185 | 55.4 | 57.5 |
| Prestvannet | 262 | 217 | 57.8 | 56.7 |
| Ramfjord-Andersdal | 162 | 182 | 57.8 | 57.3 |
| Reinelva-Sollielva | 358 | 315 | 56.3 | 56.3 |
| Sentrum | 193 | 197 | 58.4 | 58.9 |
| Sjømannsbyen | 286 | 278 | 59.1 | 59.4 |
| Skjelnan-Snarby | 232 | 251 | 56.8 | 58.4 |
| Slettaelva | 322 | 339 | 55.1 | 56.2 |
| Solneset | 191 | 207 | 50.2 | 50.6 |
| Stakkevollan | 434 | 331 | 56.6 | 56.3 |
| Stakkevollveien | 250 | 268 | 54.9 | 56.7 |
| Storelva | 296 | 288 | 51.4 | 52.1 |
| Strandveien | 206 | 174 | 58.4 | 58.4 |
| Tomasjord | 466 | 431 | 58.7 | 59.4 |
| Tromsdalen | 481 | 478 | 55.4 | 56.2 |
| Ullsfjorden-Sørfjorden | 140 | 174 | 60.6 | 62.1 |
| Workinnmarka | 565 | 418 | 56.1 | 56.5 |

Figure 1 shows the 36 areas of Tromsø municipality used in this paper and Table 1 reports number of people and mean age for each area. We did not have the information on ethnicity for each area. However, according to previous reports from Tromsø municipality on living conditions (2017), 9.3% of the population in Tromsø are non-western immigrants (Eastern Europe, Asia, Africa, and Latin America). The highest number of immigrants are in the areas of Sentrum, Gimle and Stakkevollveien, with 22.8%, 19.4% and 18.5% non-western immigrants, respectively (Figure 2) (Tromsø Kommune, 2019).


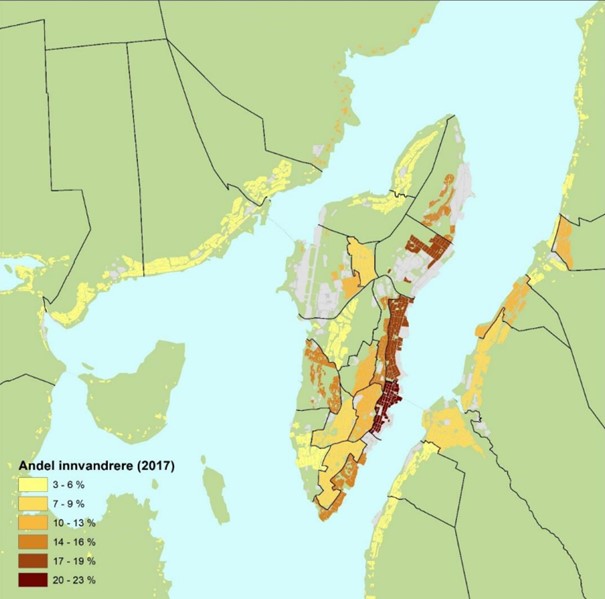


**Proportion of immigrants (2017)**

**Figure 2: Percentage of non-western immigrants according to area (Tromsø Kommune, 2019)**

- 1. **Sensitivity analysis**

We repeated the main analysis of BMI in women to see if the addition of percentage of non-western immigrants to the models would change the findings unadjusted for this covariate. Data on percentage of immigrants were taken from previous reports from Tromsø municipality on living conditions (2017) (Tromsø Kommune, 2019).

**Table 2.** Associations between area-level socio-economic status (exposure) and lifestyle behaviors (mediators) and as main effects or effects moderated by age (n=19415) in women. The Tromsø Study 2015-2016.

| **Mediators** | **Main effects not moderated by age** | **Effects moderated by age** | | |
| --- | --- | --- | --- | --- |
|  | OR (95% CI) | OR (95% CI) | | |
| Smoking (ref: Never)  Previous*  Occasional*  Current* | **-** | -1 SD | Mean age | +1 SD |
|  |  | 0.98 (0.89, 1.08)  **1.46 (1.05, 2.02)**  **0.72 (0.61, 0.85)** | **0.86 (0.80, 0.92)**  1.05 (0.85, 1.30)  **0.59 (0.52, 0.68)** | **0.75 (0.68, 0.83)**  **0.76 (0.60, 0.96)**  **0.49 (0.41, 0.57)** |
| Alcohol (ref: 0 units)  1-2 units*  3-4 units*  5 or more* | - | **1.79 (1.55, 2.08)**  **1.93 (1.60, 2.33)**  **2.00 (1.31, 3.06)** | **1.63 (1.40, 1.90)**  **1.52 (1.28, 1.80)**  1.32 (1.0, 1.73) | **1.49 (1.17, 1.88)**  1.19 (0.92, 1.53)  0.88 (0.65, 1.18) |
| Snuff (ref: Never)  Previous*  Occasional*  Current* | **1.27 (1.04, 1.55)**  0.69 (0.44, 1.07)  0.98 (0.80, 1.20) | No significant interaction with age | - | - |
| Physical activity (ref: Sedentary)  Light activity*  Moderate-to-vigorous activity* | **-** | **1.13 (1.02, 1.26)^a^** | **1.32 (1.21, 1.45)^a^** | **1.54 (1.38, 1.72)^a^** |

*All models are adjusted for age, non-western immigrants and individual-level socio-economic status and accounted for area-level clustering; All effects are estimated per unit increase in ASES; OR=odds ratio; CI=confidence interval; SD=standard deviation; B=regression coefficient; ref= reference category; proportional odds assumption was tested to see if the variable is nominal or ordinal; a=Treated as an ordinal variable (therefore only one value)*

**Nominal mediator, using generalized additive mixed models with multinominal variance*

**Table 3.** Results of mediation analysis for BMI in women: total, direct, and indirect effects of area-level socio-economic status on BMI (n=10102). The Tromsø Study 2015-2016.

| **Effects** | **BMI** |
| --- | --- |
|  | B (95% CI) |
| Total effect^a^  Association at:*  -1 SD  Mean age  +1 SD | **-0.94 (-1.17, -0.71)**  No significant interaction with age |
| Direct effect^b^  Association at:*  -1 SD  Mean age  +1 SD | **-0.85 (-1.02, -0.69)**  No significant interaction with age |
| Indirect effect, combined^c^  PME^d^ | **-0.11(-0.18, -0.05)**  0.12 (0.05, 0.19) |
| Exposure-adjusted effects of mediators: |  |
| Smoking (ref: Never)  Previous  Association at:*  -1 SD  Mean age  +1 SD  Occasional  Association at:*  -1 SD  Mean age  +1 SD  Current  Association at:*  -1 SD  Mean age  +1 SD | Mediation  **0.47 (0.26, 0.68)**  -0.11 (-0.63, 0.40)  **-0.91 (-1.22, -0.61)** |
| Alcohol (ref: 0 units)  1-2 units  Association at:*  -1 SD  Mean age  +1 SD  3-4 units  Association at:*  -1 SD  Mean age  +1 SD  5 or more  Association at:*  -1 SD  Mean age  +1 SD | Mediation  **-0.47 (-0.88, -0.07)**  0.22 (-0.22, 0.66)  **1.33 (0.70, 1.96)** |
| Snuff (ref: Never)  Previous  Occasional  Current | No mediation  0.07 (-0.51, 0.66)  0.07 (-1.24, 1.39)  -0.36 (-0.93, 0.21) |
| Physical activity (ref: Sedentary)  Light activity  Association at:*  -1 SD  Mean age  +1 SD  Moderate-to-vigorous activity  Association at:*  -1 SD  Mean age  +1 SD | Mediation  **-2.17 (-2.45, -1.89)**  **-3.41 (-3.74, -3.09)** |

*BMI=body mass index; CI=confidence interval; SD=standard deviation; B=regression coefficient; OR=odds ratio; PME=proportion of mediated effect.*

*^a^Association between exposure and outcome: main effect presented when age is not a significant moderator; age-specific associations presented when age was a significant moderator; ^b^Association between exposure and outcome not explained by mediators; ^c^Association between exposure and outcome through mediators (dietary variables not included); ^d^The proportion of the total effect mediated by mediator(s). All models were adjusted for age non-western immigrants and individual-level SES and accounted for area-level clustering. *if significant interaction with moderator (age)*

Tables 2 and 3 show that there was no change in the main findings when percentage of non-western immigrants was added as a covariate to the models.

**1.2 Spatial autocorrelation**

**Table 4. Result from Moran’s I to test spatial autocorrelation for residuals**

|  | **BMI** | | **Total/HDL cholesterol** | | **Waist circumference** | | **Hypertension** | | **Diabetes** | |
| --- | --- | --- | --- | --- | --- | --- | --- | --- | --- | --- |
|  | Women | Men | Women | Men | Women | Men | Women | Men | Women | Men |
| **Observed** | 0.0001 | 0.0001 | 0.0002 | -1.9 | 0.00003 | 0.0001 | 0.00006 | 0.00008 | -0.0003 | -0.00006 |
| **Expected** | -0.00009 | -0.0001 | -000009 | -0.0001 | -000009 | -0.0001 | -000009 | -0.0001 | -0.0001 | -0.0001 |
| **Stahdered deviation** | 0.0001 | 0.0001 | 0.0001 | 0.0001 | 0.0001 | 0.0001 | 0.0001 | 0.0001 | 0.0001 | 0.0001 |
| **p-value** | 0.10 | 0.09 | **0.006** | 0.51 | 0.31 | 0.09 | 0.18 | 0.17 | 0.21 | 0.70 |

Table 4 shows the Moran’s I test results for spatial autocorrelation of regression model residuals. Here, we can see that *p*-values were not significant for all the outcome variables except for cholesterol in women. The non-significant *p*-values indicate that there was insufficient evidence of spatial autocorrelation in the regression model residuals.

1. **Neighborhood characteristics and their pathways**

Health policy and availability of health related resources

- Prevention
- Availabilty and quality of health care

Health behavior

- Smoking
- Alcohol
- Diet
- Physical activity

Social environment

- Safety
- Cohesion
- Norms and values

Mortality

Socioeconomic and ethnic composition of inhabitants

Disease

Physical environment

- Access, availability, mix and quality of retailers, built environment and nature
- Infrastructure
- Aesthetics

Neighborhood perception

- Wellbeing
- Opportunities

Physical quality

- Pollution
- Noise
- Housing quality

**Figure 3:** Neighborhood characteristics and their pathways to inequalities in disease and mortality (Meijer, 2013)

1. **Direct acyclic graph (DAG)**


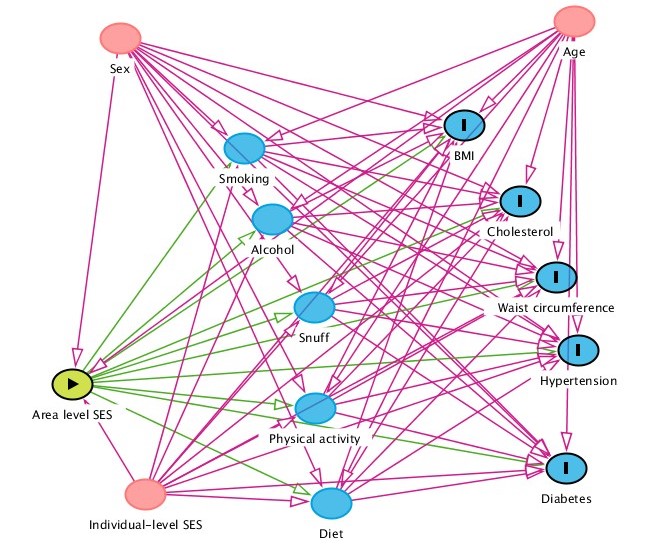


**Figure 4:** Directed Acyclic Graph (DAG) illustrating exposures, outcomes, mediators, and confounders.

**References:**

Hopstock, L.A., Løvsletten, O., Johansen, H., Tiwari, S., Njølstad, I., & Løchen, M.L. (2019). Folkehelserapport. Den sjuende Tromsøundersøkelsen 2015-16. "Public health Report. The seventh Tromsø Study". Tromsø: UiT Norges Arktiske Universitet.

Meijer, M. (2013). Neighbourhood Context and Mortality: An Overview. In Christina Stock, & Anne Ellaway (Eds.), *Neighbourhood Structure and Health promotion* pp. 11-37). New York: Springer.

Tromsø Kommune. (2019). Levekår i Tromsø: Geografisk fordeling. "living conditions report".
